# Screening in Graphene: Response to External Static Electric Field and an Image-Potential Problem

**DOI:** 10.3390/nano11061561

**Published:** 2021-06-13

**Authors:** Vyacheslav M. Silkin, Eugene Kogan, Godfrey Gumbs

**Affiliations:** 1 Donostia International Physics Center (DIPC), Paseo de Manuel Lardizabal 4, E-20018 San Sebastián, Basque Country, Spain; 2 Departamento de Polímeros y Materiales Avanzados: Física, Química y Tecnología, Facultad de Ciencias Químicas, Universidad del País Vasco (UPV-EHU), Apdo. 1072, E-20080 San Sebastián, Basque Country, Spain; 3 IKERBASQUE, Basque Foundation for Science, E-48011 Bilbao, Basque Country, Spain; 4 Department of Physics, Jack and Pearl Resnick Institute, Bar-Ilan University, Ramat-Gan 52900, Israel; Eugene.Kogan@biu.ac.il; 5 Department of Physics and Astronomy, Hunter College of the City University of New York, 695 Park Avenue, New York, NY 10065, USA; ggumbs@hunter.cuny.edu

**Keywords:** graphene, electric field, valence charge density, image potential, image-plane position, image-potential states

## Abstract

We present a detailed first-principles investigation of the response of a free-standing graphene sheet to an external perpendicular static electric field *E*. The charge density distribution in the vicinity of the graphene monolayer that is caused by *E* was determined using the pseudopotential density-functional theory approach. Different geometries were considered. The centroid of this extra density induced by an external electric field was determined as $z_{\mathrm{im}}$ = 1.048 Å at vanishing *E*, and its dependence on *E* has been obtained. The thus determined $z_{\mathrm{im}}$ was employed to construct the hybrid one-electron potential which generates a new set of energies for the image-potential states.

## 1. Introduction

The numerous properties of graphene have been intensively investigated after its experimental realization. Thousands of papers on this material were published. However, there still remains a simple unanswered question regarding the way in which the induced charge density is distributed in the vicinity of a graphene monolayer when an external electric field is applied to the graphene sheet. This topic was addressed, to some degree, by considering the problem of screening of the electric field induced by point charges in graphite [1,2,3,4,5]. Specifically, the in-plane distribution of the induced charge has been actively discussed [5,6,7,8,9,10]. As for the charge distribution in the direction perpendicular to the plane of carbon atoms, it was considered as being localized on it [5].

The perpendicular charge distribution was studied by considering two- and multi-layer graphene films [11,12,13], though to the best of our knowledge, not for monolayer graphene. Moreover, regarding the question around the location of its center of mass with respect to the carbon atoms position, we are unaware of such work for a graphene film of any thickness. As a matter of fact, this question is important since, for instance, the position of the centroid of the induced density determines the so-called image-plane position $z_{\mathrm{im}}$, (here we define the *z* axis as pointing in the direction perpendicular to the carbon atoms basal plane) that is a “real position” of a solid surface for many phenomena occurring there. It determines a “physical” position of a metal surface when an external perturbation is applied. This problem was widely studied in the case of metal surfaces. In general, this “real” surface position is different from the spatial localization of the top atomic layer or a geometrical crystal edge, staying towards the vacuum side [14,15,16,17,18].

It is usually assumed for a quasi two-dimensional (2D) system that the excess charge is confined within an infinitesimally thin 2D layer [5,19]. Certainly, this assumption is reasonable if the relevant distance largely exceeds the atomic scale. However, it is critical to take into consideration what occurs on the atomic scale. For instance, if one intends to construct a capacitor by adopting graphene sheets, it would be helpful to determine its “physical size” which defines its electrical properties and may be different from the geometrical distance between two graphene layers. Addionally, determination of the spatial localization of the charge induced by an external electric field can be important in understanding the phenomena occurring in field-effect transistors based on 2D materials [20,21,22].

Knowledge of the position of the center of mass of the induced charge density is important in many fields of surface science. Thus, it determines the reference plane for the image-potential felt by an external charge placed in front of a surface. If this charge is an excited electron with energy below the vacuum level, it can be trapped by this image potential in a state belonging to an infinite Rydberg -like series [23,24]. The members of this series are referred to as image-potential states (IPSs).

In the previous work devoted to the IPSs in graphene, it was assumed [25,26] that $z_{\mathrm{im}}$ is located at the carbon atom plane, which seems reasonable owing to the mirror symmetry of the system. Consequently, all the quantum states should be symmetric or anti-symmetric with respect to the z=0 plane. As a result, a double Rydberg -like series of IPSs was predicted [25] to exist in a free-standing graphene monolayer since two surfaces are separated by a single atomic layer of matter only.

Up to now, IPSs for a free-standing graphene were not studied experimentally. On the other hand, numerous measurements were performed on the graphene supported on various metallic or semiconducting substrates. Usually, the interface distance between the graphene sheet and the surface atomic layer is such that the conventional single Rydberg series of a whole system is observed. Thus, in the graphene/metal systems where the graphene atomic layer is placed closer to the substrate, only a single series of IPSs was observed [27,28,29,30,31,32,33,34,35,36]. Nevertheless, there are cases where the distance separating the graphene and the top surface atomic layer is sufficiently large so as to realize the two lowest members of the graphene double-IPS series. In scanning-tunneling microscopy (STM) measurements, evidence for the Stark-shifted first two members (symmetric and antisymmetric ones) of this series was reported in the Gr/SiC(0001) system [37,38]. These states were also clearly observed in two-photon photoemission spectroscopy experiments [39]. However, in the same system, the splitting of the IPS series was not confirmed in the Ref. [40]. In the very recent experimental paper, the arguments in favor of the splitting were presented [41].

For a description of the IPSs in the graphene/substrate systems, a number of potentials have been developed. Indeed, an accurate description of IPSs is a challenge since the conventional density-functional theory (DFT) calculations do not accurately account for a correct long-range interaction in front of solid surfaces. One of the approaches consists of constructing the nonlocal van der Waals functional [42]. Although it does not yield the correct image potential behavior at long distances away from the 2D sheet, it improves the IPS description. Another input employing a conventional DFT scheme based on the local-density approximation (LDA) consists of the construction of a hybrid potential with the same computational cost. Some others use totally model potentials [26,35,43]. Since the binding energies of IPSs are sensitive to the long-range behavior of an effective potential, a key point is the image-plane position $z_{\mathrm{im}}$ with respect to the carbon atom plane. Upon construction of the model potential in the Ref. [43], the fitting procedure gave $z_{\mathrm{im}}=0.99$ Å. This is significantly different from $z_{\mathrm{im}}=0$ assumed in other publications [25,26].

Our goal in this work is to determine the $z_{\mathrm{im}}$ value for free-standing monolayer graphene from the direct DFT calculations of redistribution of its valence charge density upon application of an external electric field. Subsequently, the thus obtained $z_{\mathrm{im}}$ is employed for the construction of a new hybrid “LDA+image−tail” potential. With this potential a new set of binding energies for IPSs is obtained and compared with the previous ones.

The rest of this paper is organized as follows. In Section 2, a brief description of our calculation method and some computational details are given. In Section 3, we present our calculated results. A summary and concluding remarks are presented in Section 4.

## 2. Calculational Methods and Details

The band structure of a graphene monolayer in the absence and presence of an external electric field of varying intensity was obtained within the LDA by solving the Kohn-Sham equations employing a home-made band structure computer code [44]. We used norm-conserving Troullier-Martin pseudopotentials to describe the electron-ion interaction for the carbon ions [45]. At the iteration stage, the exchange-correlation potential was taken in the form given in the Refs. [46,47]. For the expansion of the wave functions, a plane-wave basis set with an energy cutoff of 50 Rydberg was employed. In a self-consistent procedure, the summation over wave vectors in the irreducible part of the first Brillouin zone (BZ) was performed over a 48 × 48 × 1 k mesh.

**Figure 1:**
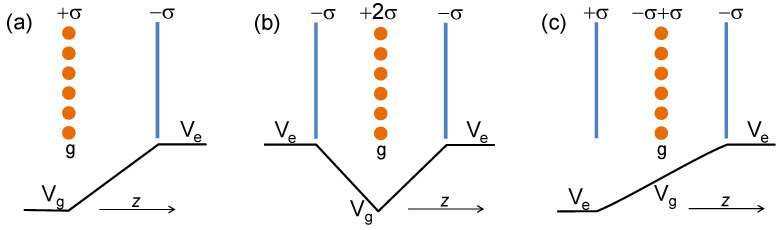
Schematic illustration of three geometries considered in this work of a graphene sheet (solid circles) interacting with an external electric charge uniformly distributed in the *x*-*y* plane with density −σ at a chosen distance in the *z* direction.In the geometry (**a**) this extra charge is placed on the right. In the case (**b**) the charges of the same signs are located on the left and right sides. Panel (**c**) illustrates the geometry when the charges of the opposite sings placed on each side.

The self-consistent procedure was realized by considering a repeated-slab geometry with the lateral lattice constant of 2.424 Å. The external electric field applied in the direction perpendicular to the graphene plane has no translation symmetry. In order to implement it in the repeated-slab geometry, we added to the Hamiltonian a term corresponding to the extra charge −σ(z) constant in the *x*-*y* plane as shown in Figure 1a. Its *z*-dependence is defined by a Gaussian with a decay length of 1 a.u. This extra charge was placed at a distance of 10 Å from the graphene plane. In order to ensure the neutrality of the system, the charge +σ was removed from the graphene system. The *z* variation of the extra potential added to the system is schematically shown in Figure 1a. One can see that in the gap between the graphene and the extra charge position, this potential varies linearly from V${}_{g}$ to V${}_{e}$ with the E=2πσ slope. The problem with such a geometry is that there is a discontinuity in the potential between the left and right sides. In order to employ the repeated-slab geometry, we double the unit cell by mirror reflection of the picture of Figure 1a and establishing the distance between the graphene sheets in 20 Å. The resulting lattice constant in the perpendicular direction is 40 Å. We performed calculations considering the electric fields applied to the graphene sheet ranging from −0.4 to 0.5 V/Å with a step of 0.1 V/Å and keeping the in-plane (1 × 1) geometry for carbon ion positions.

In other sets of calculations, we considered a geometry when the external electric field is applied from both sides of the graphene sheet as shown in Figure 1b. In this case, the unit cell contains only one graphene sheet and a lattice parameter of 20 Å is chosen. This geometry allows us to investigate the scale on which the charge density distribution established in graphene can be considered additively. On the other hand, this geometry is not suitable for the determination of the image-plane position since the resulting system is symmetric by construction. The third geometry considered in this study is schematically presented in Figure 1c. In this case, the two planes charged with +σ and −σ are located on each side of the graphene sheet that, in turn, is kept neutral. Since the total induced charge of the graphene is zero, this geometry cannot be used for the determination of the $z_{\mathrm{im}}$ position. Nevertheless, the polarization induced in the carbon atom plane by the external field can be represented by two charged planes. In such a way, each surface can be considered as the covers of the different capacitors and charged oppositely.

## 3. Calculation Results

**Figure 2:**
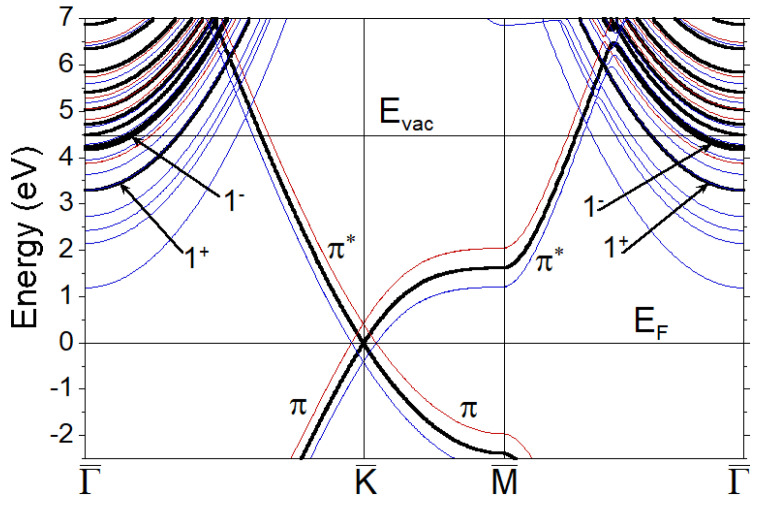
Electronic band structure of graphene when E=0 (thick black lines), 0.4 V/Å (thin red lines), and −0.4 V/Å (thin blue lines) obtained with application of the geometry of Figure 1a. The Fermi level, E${}_{F}$, is placed at zero energy. The position of the vacuum level, E${}_{\mathrm{vac}}$, is shown for the zero field. The π and $\pi^{*}$ bands are marked by corresponding symbols. The two unoccupied lowest energy states around the $\bar{\Gamma }$ point with strong localization in the vacuum are marked as $1^{+}$ and $1^{-}$ according to the Ref. [25].

The electronic structure of graphene around the Fermi level at zero external electric field is presented in Figure 2 by thick black lines. The carbon-derived bonding and antibonding π bands are marked as π and $\pi^{*}$, respectively. The two lowest energy bands above the Fermi level characterized by strong expansion into the vacuum are marked as $1^{+}$ and $1^{-}$. At energies above the vacuum level, one can notice the quantization of the bands representing a free-electron continuum due to the finite size of the vacuum interval. In the same figure, we show how the energy position of all these bands changes when the external electric field of −0.4 V/Å (blue curves) or 0.4 V/Å (red curves) is applied. One can notice that the π and $\pi^{*}$ bands experience a shift of almost the same magnitude from the bare dispersion upon changing the sign of the electric field. On the contrary, the position of the upper energy bands with a strong expansion of its wave functions into the vacuum side changes differently for opposite signs.

### 3.1. Electric Field Effects

**Figure 3:**
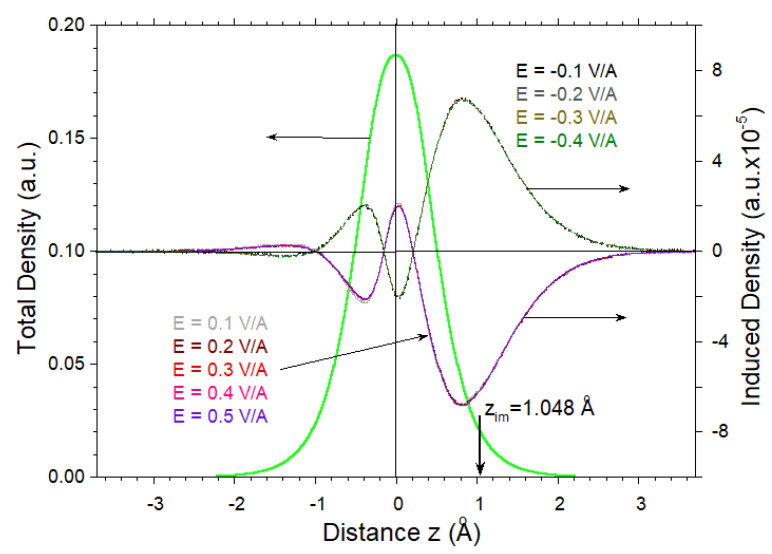
The valence charge density of graphene averaged in the *x*-*y* plane (green thick line) and induced charge densities generated by an applied electric field *E* for the color-coded values shown in the insets. The induced density for E=θ×0.1 V/Å is normalized by the value of |θ|. The origin of the *z* direction is taken as the carbon atom position. The image plane position $z_{\mathrm{im}}$ at 1.048 Å is marked by vertical arrow (the positive value is due to the application of the electric field from the right side).

**Figure 4:**
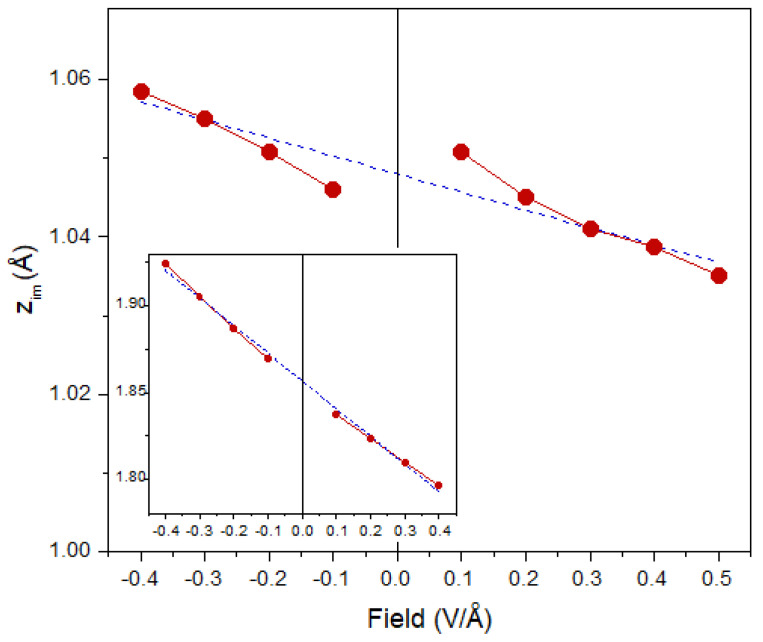
Dependence of the image plane position $z_{\mathrm{im}}\left(E\right)$ in graphene versus the electric field *E* amplitude (red circles and solid line). The linear interpolation is shown by a blue dashed line. The insert shows the way in which $z_{\mathrm{im}}\left(E\right)$ depends on *E* in the case of an Al(111) monolayer.

We have examined the way in which the induced charge density profile $n_{\mathrm{ind}}\left(r,E\right)$ varies with the strength and direction of the applied electric field *E*. Figure 3 reports $n_{\mathrm{ind}}\left(z,E\right)$ obtained by averaging $n_{\mathrm{ind}}\left(r,E\right)$ in the *x*-*y* plane for the values of *E* ranging from −0.4 to 0.5 V/Å. In order to perform a comparison, $n_{\mathrm{ind}}\left(z,E\right)$ is normalized by the amplitude of *E*. One can see that its shape deviates qualitatively from the total valence density depicted by the green solid line. This can be understood, since the total density is dominated by the σ bands that have a maximum at z=0. On the contrary, the induced density is generated mainly by π bands. Additionally, one can observe that the shape of the induced density only slightly depends on the sign and the magnitude of *E*. In general, we observe that at larger *E*, the shapes of $n_{\mathrm{ind}}\left(z,E\right)$ are almost the same. However, upon reduction of the *E* amplitude, the variations in $n_{\mathrm{ind}}\left(z,E\right)$ gradually increase (hardly noticeable in Figure 3). This has consequences in the calculated centroid of the induced charge density versus *E*, defined as

(1) $$z_{\mathrm{im}}\left(E\right)=\frac{\int z\;n_{\mathrm{ind}}\left(z,E\right)\;dz}{\int n_{\mathrm{ind}}\left(z,E\right)\;dz}$$

and presented in Figure 4. Linear interpolation gives a value of 1.048 Å for $z_{\mathrm{im}}\left(E=0\right)$. Curiously, by constructing a model potential to describe IPSs measured experimentally in graphene/substrate systems, a very close value of 0.99 Å was established for $z_{\mathrm{im}}$ in graphene monolayer [43]. A similar value was chosen for the crystal border in graphene in the Ref. [48]. We expect that the value of $z_{\mathrm{im}}$ obtained here should not be affected significantly by the presence of the substrate once the valence electronic structure of graphene is not modified strongly by the substrate. In Figure 4, one can notice that upon approaching the E=0 limit, $z_{\mathrm{im}}\left(E\right)$ starts to deviate from the linear behavior. Moreover, this deviation is different for negative and positive *E*. In the former case, $z_{\mathrm{im}}$ shifts downward, whereas in the latter case it is shifted upward. This can be explained by the fact that with reduction in the magnitude of *E*, the size of the Fermi surface shrinks and the possible calculation oscillations increase. For comparison, in the insert of Figure 4, we present the way in which $z_{\mathrm{im}}\left(E\right)$ varies with *E* in a free-standing Al(111) monolayer. Since, for Al, the Fermi surface is large because there are three valence electrons, the deviation from the linear behavior is small.

When we apply an external electric field to the graphene sheet from both sides according to the scheme depicted in Figure 1b, the induced charge density has a symmetric shape owing to the mirror symmetry. Its shape can be reproduced very well by superimposing that of Figure 3 onto a reflection of itself, thereby demonstrating that the response is additive. It means that once one knows how the electronic system of a graphene sheet responds to an external electric field applied from one side, the response to a more complex external perturbing field can be readily evaluated.

Clearly, in the symmetric case (Figure 1b), the calculated centroid of the induced charge density is placed at z=0. However, knowing that each side responds independently to external electric fields applied from both the respective sides, the resulting charge distribution can be described in the electrostatic limit by two planes charged with the same signs and located at $z=-z_{\mathrm{im}}$ and $z=z_{\mathrm{im}}$. We believe that this picture should hold for a case when the valence electronic system is perturbed in a photoemission experiment, for example. In this case, an excited electron is promoted above the Fermi level. If its kinetic energy is lower than the work function, it can be trapped in the discrete IPSs whose number is two times larger than in the conventional Rydberg series of the hydrogen atom [25].

In the case of the geometry described in Figure 1c, the charge redistribution in the neutral graphene caused by placing it inside a capacitor can be represented at the electrostatic level by two charged planes with the opposite signs located at $z=-z_{\mathrm{im}}$ and $z=+z_{\mathrm{im}}$. Moreover, we found that the shape of the calculated induced density is also reproduced very well by employing the charge density distributions obtained for the positive and negative *E*s reported in Figure 1a. Since the calculated induced densities and the fitting results are very similar we do not include such a figure.

### 3.2. Image-Potential States

In our numerical calculations devoted to IPSs, the perpendicular lattice constant was increased up to 80 Å which allowed us to obtain convergent energies for the six lowest-energy members of the series. As it was mentioned previously, IPSs cannot be properly described with the use of conventional DFT calculations since the long-ranged image-potential on the vacuum side is not reproduced correctly. Additionally, the tight-binding methods are not inherently desired for its description [49,50]. Indeed, such states are a result of screening by the valence electron system of an external point charge placed in front of a system. This many-body information is not contained in the one-particle DFT Hamiltonian. In order to overcome this problem, maintaining the computational cost at the DFT level, we constructed a hybrid “LDA+image−tail” potential V(r) which replaces the LDA local exchange-correlation potential term $V_{\mathrm{xc}}\left(r\right)$ in the DFT Hamiltonian. At |z| smaller than a certain $z_{o}$ value this potential coincides with $V_{\mathrm{xc}}\left(r\right)$. For $|z|>z_{o}$, it has the following form:

(2) $$V\left(r\right)=-\frac{1-A\left(x,y\right)\cdot e^{-\lambda \left(x,y\right)\cdot |z-\mathrm{sgn}\left(z\right)\cdot z_{\mathrm{im}}|}}{4|z-\mathrm{sgn}\left(z\right)\cdot z_{\mathrm{im}}|}.$$

The parameters A(x,y) and λ(x,y,) are defined from the smoothness conditions for V(r) and its derivative at the matching planes $\left|z\right|=z_{o}$. In this work, these parameters depend on the *x* and *y* coordinates, since $V_{\mathrm{xc}}\left(x,y,z\right)$ still has a small corrugation at the matching plane. The only parameter left is $z_{o}$ which is unknown. In the following, we present results for three values of $z_{o}$ to show the sensitivity of the image-potential state energies to it.

**Figure 5:**
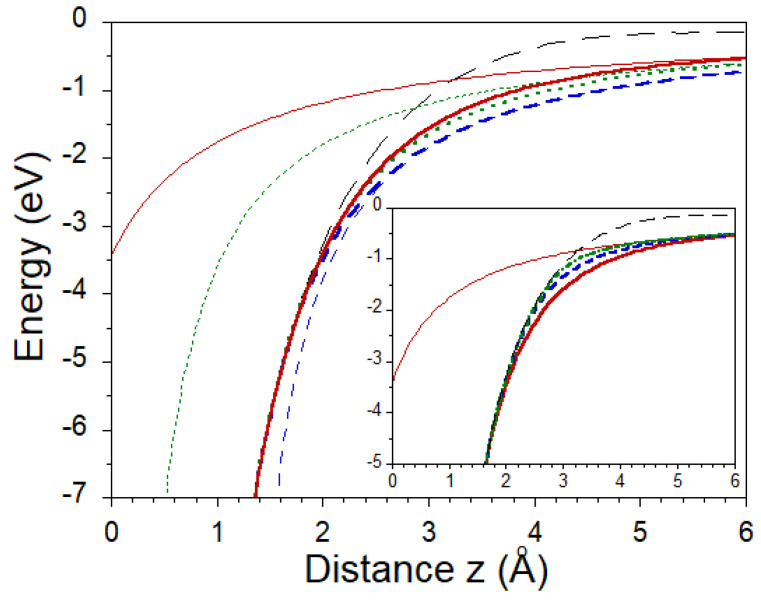
The LDA potential averaged in the *x*-*y* plane as a function of the *z* distance is shown by the thin dashed line. Hybrid “LDA+image−tail” potentials for $z_{\mathrm{im}}$ = 0, 1.048, −1.048 Å with the matching plane at $z_{0}$=1.6 a.u. are presented as thick dotted, dashed, and solid lines, respectively. The corresponding bare image potentials are shown by thin dotted, dashed, and solid lines, respectively. Insert: Hybrid “LDA+image−tail” potentials constructed for $z_{\mathrm{im}}$ = −1.048 Å with the matching planes $z_{0}$ = 1.6, 2.1, and 2.6 Å are represented by thick solid, dashed, and dashed-dotted lines, respectively.

In Figure 5, the thick dashed line shows the hybrid “LDA+image−tail” potential averaged in the *x*-*y* plane constructed with $z_{\mathrm{im}}$ = 1.048 Å and $z_{o}$ = 1.6 Å. One can see how at distances *z* larger than $z_{o}$ it evolves from the averaged LDA potential (thin long-dashed line) to the image-potential defined as $-1/4(z-z_{\mathrm{im}})$ (thin dashed line). Notice that the potentials we construct here and employ for the band structure calculations are symmetric according to the z=0 plane. Here, we show its behavior for positive *z* only. For comparison, in Figure 5 by thick dotted line, we show the hybrid potential constructed for $z_{\mathrm{im}}$ = 0 and $z_{o}$ = 1.6 Å of the Ref. [25]. One can see that the hybrid potential constructed with $z_{\mathrm{im}}$ = 1.048 Å is noticeably lower for *z* larger than $z_{o}$. This results in larger binding energies of IPSs. This is confirmed by the values obtained at the center of the BZ as reported in Table 1. One can see that the binding energy of the lowest-energy symmetric $1^{+}$ state increases from the 1.47 eV of the Ref. [25] to 1.58 eV here. Almost the same change is experienced by the antisymmetric $1^{-}$ state. For the states with larger numbers, this shift is notably smaller. Certainly, as *n* is increased this difference is gradually reduced. With $z_{\mathrm{im}}$ = 1.048 Å by employing $z_{o}$ larger than 1.6 Å we encountered a problem with the construction of the hybrid potential. Beyond this value for $z_{o}$, the two matching conditions for the hybrid potential cannot be fulfilled since the image potential with $z_{\mathrm{im}}$ = 1.048 Å is located too far away on the right-hand side of the LDA potential, as seen in Figure 5. Notice that the downward shift of the IPSs is observed over a whole BZ. Nevertheless, this does not significantly affect the interaction of IPSs with the scattering resonances [51] around the $\bar{K}$ point.

**Figure 6:**
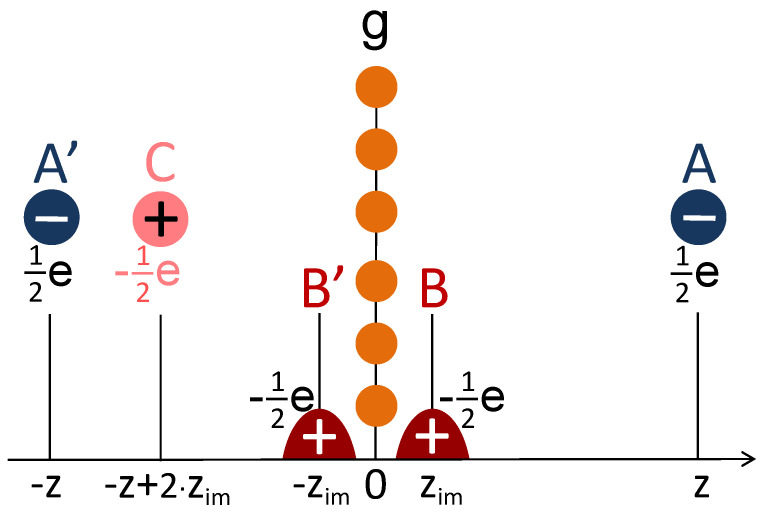
Schematic illustration of the charges created in the vicinity of a graphene monolayer. The carbon atom plane is located at z=0. The charge distribution in an image-potential state with the centers of gravity on the positive and negative sides according to the graphene plane located at *z* and −z are represented by two point charges as shown by blue circles A and A${}^{\prime }$. The positive charge density generated in graphene in response to this external perturbation is represented by red areas B and B${}^{\prime }$ centered at $z_{\mathrm{im}}$ and $-z_{\mathrm{im}}$, respectively. As a result, the negative point charge A interacts with its own image charge C, negative charge A${}^{\prime }$, and positive charge B${}^{\prime }$.

The approach described above for construction of a hybrid “LDA+image−tail” potential is an adoption of the conventional image-potential picture employed for solid surfaces [52]. It can also be safely applied for sufficiently thick films as well. However, in a film consisting of just a single atomic layer, the situation might be different. In such a system, in a photoemission experiment, an excited electron can occupy a quantum state with the charge density symmetrical with respect to the atom plane, contrary to what occurs for solids where only a single surface is involved. The presence of two independent surfaces was indeed taken into account in our model presented above. Nevertheless, let us consider the situation from another point of view by applying a simple image-potential picture in a different way. In this case, we will replace an excited electron with some spatial charge density distribution by two point charges having $\frac{1}{2}$e located at distances *z* and −z as denoted by A and A${}^{\prime }$, respectively, in Figure 6. In the graphene sheet, these two point charges create the screening charges B and B${}^{\prime }$ whose centers of gravity are located at $z_{\mathrm{im}}$ and $-z_{\mathrm{im}}$, respectively. The spacial arrangement of these screening charges should be such to ensure efficient screening and avoid any charge current. Assuming the distance *z* is large, let us account for the interaction between the charge A with real charges A${}^{\prime }$, B, and B${}^{\prime }$. The interaction of A with the charge B can be replaced by interaction with its image point charge C with positive sign located at $-z+2z_{\mathrm{im}}$, like it occurs at a metal surface. The interaction with a point charge A${}^{\prime }$ is obviously a Coulomb-like one. However, the interaction with a charge B${}^{\prime }$ is not obvious. The space distribution of this part of the total screening charge is such as to screen the point charge A${}^{\prime }$. Therefore, since *z* is large, for a point charge A, it can be considered as a point charge located at $-z_{\mathrm{im}}$. Counting all these three contributions at the first order in 1/z, the resulting potential takes the form $V\left(z\right)=-1/4|z+z_{\mathrm{im}}|$, that is, it looks like a charge A interacting with a point charge located at $z=-z_{\mathrm{im}}$. A factor of four in the denominator is due to a fractionally charged electron with half its charge representing the A and B${}^{\prime }$ charges. Clearly, this latter model might be reasonable for IPSs with high numbers *n*. However, it may not be good for $n=1^{+}$ since the maximum of its wave function is localized [25] around 2 Å, that is, being very close to the $z_{\mathrm{im}}$ = 1.048 Å position.

Based on this picture, we constructed a hybrid “LDA+image−tail” potential for which $z_{\mathrm{im}}$ is placed at −1.048 Å. This potential with the matching plane at $z_{o}$ = 1.6 Å is shown as the thick solid line in Figure 5. Our calculated energies for the five lowest image-potential states are reported in Table 1. Comparing them with those obtained for $z_{\mathrm{im}}$ = 0 and $z_{\mathrm{im}}$ = 1.048 Å we observe a significant reduction of the binding energies, especially for n=1. Varying $z_{o}$, we do not encounter problems with construction of the hybrid potential, contrary to the situation with $z_{\mathrm{im}}$ = 1.048 Å. For completeness, in Table 1, we report the image-potential energies obtained with $z_{o}$ = 2.1 and 2.6 Å as well. The respective hybrid potentials are reported in the insert of Figure 5. One can see that the effect of the variation in the potential caused by changing $z_{o}$ on the states $n=1^{+}$ and $n=1^{-}$ is substantial. Thus, for the lowest-energy image-potential state the binding energy may vary from 1.58 to 1.27 eV depending on the values of $z_{\mathrm{im}}$ and $z_{o}$. Indeed, one can see how by increasing $z_{o}$, the value for the state $1^{+}$ is approaching the energy of 1.17 eV for the surface state [53,54] obtained in the LDA calculation [25]. However, for the state $1^{-}$, it is not the case.

We believe, the measurements of energies of free-standing graphene will provide important information on its screening properties. It may contribute to establishing a detailed picture of what is going on there due to an external perturbation. So far, all the experiments on IPSs were performed on supported graphene. Sensitivity of the image-potential states to the environment where graphene was kept was significant. Our findings point out that they can also provide important information about free-standing graphene screening properties.

## 4. Conclusions

In this theoretical study we have reported the detailed charge density distribution produced in free-standing graphene by external static electric fields with three geometries. The image-plane position was established. Surprisingly, it is rather large, located at 1.048 Å outside the carbon atom plane. Using this information, we constructed a new potential felt by an electron excited to the image-potential states. We checked several kinds of such a potential, demonstrating sensitivity of the energies of the lowest image-potential states to the details of this potential. It would be of interest to obtain the experimental information, such as from the photoemission spectroscopy, on the image-potential state energies for free-standing graphene. We believe that the experimentally determined image-potential energies will be extremely helpful for development of a more detailed picture for the graphene potential and how it reacts to the external perturbation on the atomic scale.

Our data on $z_{\mathrm{im}}$ gives support to the value used for construction of an effective potential in the graphene/substrate systems [43]. Such potentials can be developed for the study of IPSs and interface states in a large class of molecular layers with the π-π interaction similar to graphene [55,56,57,58,59]. Moreover, the information on the image-plane position can be useful for the construction of effective potentials in the systems with more complex geometries like fullerens and nanotubes, where the nearly-free states and the super-atomic orbitals, a subject of intense ongoing research, are inherently linked to IPSs in a flat graphene layer [60,61,62,63,64,65,66,67]. We believe that such a study as ours will not only be restricted to the carbon atoms case, since image-potential states can be realized in many other quasi-2D systems of current interest, like phosphorene, silicene and germanene [68], borophene [69], MXenes [70,71,72], and molecular overlayers on graphene [73].

## Figures and Tables

**Table 1:** Binding energies (in eV) of the image-potential states in graphene obtained with the hybrid potentials constructed with $z_{o}$ = 1.6 Å and $z_{\mathrm{im}}$ = 1.048 and $z_{\mathrm{im}}$ = 0 Å. In the case of $z_{\mathrm{im}}$ placed at −1.048 Å the energies are obtained for three values of the matching plane position $z_{o}$. Last line presents the values of the states obtained in the LDA calculation [25].

$z_{\mathrm{im}}\left(Å\right)$	$z_{o}\left(Å\right)$	$1^{+}$	$1^{-}$	$2^{+}$	$2^{-}$	$3^{+}$
1.048	1.6	1.58	0.84	0.29	0.21	0.12
0	1.6	1.47	0.72	0.25	0.19	0.11
−1.048	1.6	1.43	0.64	0.21	0.16	0.10
	2.1	1.30	0.52	0.19	0.15	0.11
	2.6	1.27	0.49	0.20	0.15	0.10
LDA		1.17	0.25	-	-	-

## Abbreviations

The following abbreviations are used in this manuscript:

2D	Two-dimensional
IPS	Image-potential state
STM	Scanning-tunneling microscopy
DFT	Density-functional theory
LDA	Local-density approximation
BZ	Brillouin zone
